# Determination of the autophagic flux in murine and human peripheral blood mononuclear cells

**DOI:** 10.3389/fcell.2023.1122998

**Published:** 2023-03-13

**Authors:** Sophia Walter, Tobias Jung, Catrin Herpich, Kristina Norman, Olga Pivovarova-Ramich, Christiane Ott

**Affiliations:** ^1^ Department of Molecular Toxicology, German Institute of Human Nutrition Potsdam-Rehbruecke, Nuthetal, Germany; ^2^ DZHK (German Center for Cardiovascular Research), Partner Site Berlin, Berlin, Germany; ^3^ Department of Nutrition and Gerontology, German Institute of Human Nutrition Potsdam-Rehbruecke, Nuthetal, Germany; ^4^ Department of Geriatrics and Medical Gerontology, Charité Universitaetsmedizin Berlin, Corporate Member of Freie Universitaet Berlin and Humboldt-Universitaet zu Berlin, Berlin, Germany; ^5^ Institute of Nutritional Science, University of Potsdam, Nuthetal, Germany; ^6^ Department of Molecular Nutritional Medicine, German Institute of Human Nutrition Potsdam-Rehbruecke, Nuthetal, Germany; ^7^ Department of Endocrinology, Diabetes and Nutrition, Charité-Universitaetsmedizin Berlin, Corporate Member of Freie Universitaet Berlin, Humboldt-Universitaet zu Berlin, and Berlin Institute of Health, Berlin, Germany; ^8^ German Center for Diabetes Research (DZD), Neuherberg, Germany

**Keywords:** aging, cardiomyocytes, lysosomes, concanamycin A, LC3, NZO, sequestome-1, TFEB

## Abstract

The autophagy lysosomal system (ALS) is crucial for cellular homeostasis, contributing to maintain whole body health and alterations are associated with diseases like cancer or cardiovascular diseases. For determining the autophagic flux, inhibition of lysosomal degradation is mandatory, highly complicating autophagy measurement *in vivo*. To overcome this, herein blood cells were used as they are easy and routinely to isolate. Within this study we provide detailed protocols for determination of the autophagic flux in peripheral blood mononuclear cells (PBMCs) isolated from human and, to our knowledge the first time, also from murine whole blood, extensively discussing advantages and disadvantages of both methods. Isolation of PBMCs was performed using density gradient centrifugation. To minimize changes on the autophagic flux through experimental conditions, cells were directly treated with concanamycin A (ConA) for 2 h at 37°C in their serum or for murine cells in serum filled up with NaCl. ConA treatment decreased lysosomal cathepsins activity and increased Sequestosome 1 (SQSTM1) protein and LC3A/B-II:LC3A/B-I ratio in murine PBMCs, while transcription factor EB was not altered yet. Aging further enhanced ConA-associated increase in SQSTM1 protein in murine PBMCs but not in cardiomyocytes, indicating tissue-specific differences in autophagic flux. In human PBMCs, ConA treatment also decreased lysosomal activity and increased LC3A/B-II protein levels, demonstrating successful autophagic flux detection in human subjects. In summary, both protocols are suitable to determine the autophagic flux in murine and human samples and may facilitate a better mechanistic understanding of altered autophagy in aging and disease models and to further develop novel treatment strategies.

## 1 Introduction

Macroautophagy, hereafter referred to as autophagy, is a catabolic process which delivers parts of the cytosol, as a reaction to different stressors such as starvation, infection or hypoxia, into the lysosome for degradation (Dikic and Elazar, 2018). By degrading and recycling of dysfunctional proteins or damaged cell compartments, the autophagy lysosomal system (ALS) contributes to ensure cellular energy homeostasis, function and survival of the cell (Gross and Graef, 2020). Autophagy can support regeneration, for example, of cardiac tissue after myocardial infarction (Dong et al., 2019), but can also promote tumor growth (Li et al., 2020). Impaired autophagy is observed in neurodegenerative diseases such as Alzheimer and Parkinson disease but also during normal aging, contributing to increased accumulation of protein aggregates (Leidal et al., 2018; Park et al., 2020). Lifespan increasing interventions such as caloric restriction, physical activity or rapamycin treatment often rely on improved autophagy (Dikic and Elazar, 2018; Klionsky et al., 2021a).

To investigate autophagy, it is recommended to measure the autophagic flux, describing a dynamic process that includes autophagosome formation, transport into lysosomes and degradation by lysosomal enzymes (Klionsky et al., 2021b). Since autophagic marker proteins are also degraded by lysosomal enzymes, expression of autophagic marker proteins should always be analyzed with and without lysosomal inhibitors to obtain the degradation rate. Therefore, compounds such as bafilomycin A1 or concanamycin A (ConA) are often used, as they are blocking lysosomal V-ATPases (Huss et al., 2002). Consequently, lysosomal pH increases and leads to the inhibition of lysosomal enzyme activity, resulting in an accumulation of autophagic cargo. This can be measured by an increase of autophagic flux proteins, such as Microtubule-associated proteins 1A/1B light chain 3-II (LC3-II) or Sequestome 1 (SQSTM1), compared to samples without lysosomal inhibitor (Yoshii and Mizushima, 2017; Klionsky et al., 2021b). Transcription of autophagy and lysosome associated genes is among others regulated by transcription factor EB (TFEB), translocating into the nucleus during lysosomal stress conditions, such as starvation (Settembre et al., 2012; Zhitomirsky et al., 2018).

For monitoring the autophagic flux *in vivo*, non-invasive methods are limited, as a lysosomal inhibitor has to be applied. To address this issue, we have chosen peripheral blood mononuclear cells (PBMCs) as a cell model, since PBMCs are easily to isolate and treat. PBMCs are also an optimal tool for our analyses since they have shown to be affected by aging (Busquets-Cortés et al., 2018; McCormick et al., 2018) and disease such as hypertension, lupus or cancer (Liew et al., 2006; Mello et al., 2012) and react to environmental changes like exercise (McCormick et al., 2019), smoking (Wieczfinska et al., 2018) and diet interventions (Lowry et al., 2019).

Until now, most studies in PBMCs focused on basal expression of autophagy marker proteins without any inhibitor treatment (Mejías-Peña et al., 2016; Wu et al., 2018; Huang et al., 2019; Lowry et al., 2019; McCormick et al., 2019) or used PBMCs, which were treated with lysosomal or autophagy inhibitors in complete cell culture media (Phadwal et al., 2012; François et al., 2015; Pietrocola et al., 2017; McCormick et al., 2018; Papagiannakis et al., 2019; Alsaleh et al., 2020). Only Bensalem et al. presented a protocol considering the physiological environment of the cells, directly treating whole blood samples with chloroquine for 1 h after blood collection (Bensalem et al., 2021). However, these studies were merely performed using human PBMCs lacking results in other species. Therefore, we aimed to establish a protocol for the determination of the autophagic flux that reflects the *in vivo* conditions as close as possible, using PBMCs isolated from murine and human blood samples. Even if different studies exist describing autophagy measurements in human samples, protocols provided herein enable fast and easy isolation of PBMCS from human and murine blood samples and the detection of the autophagic flux, by directly treating the cells with lysosomal inhibitor ConA for 2 h in their respective serum.

Short-term incubation of PBMCs with ConA in human serum or in murine serum filled up with NaCl conducted to an inhibition of lysosomal activity and autophagic flux in both species. To investigate, if the autophagic flux in PBMCs is affected by aging, we isolated PBMCs from young and old mice. In a second step, we compared age-related effects on autophagic flux in PBMCs to alterations in cardiomyocytes isolated from the same animal. In PBMCs, an aging effect on autophagic flux was determined, which was not present in isolated cardiomyocytes from the same mice. Furthermore, we included a limitations and advantages section, discussing benefits and disadvantages of all analyses. In summary, this study provides an easy-to-use method for determination of the autophagic flux in murine and human PBMCs, the latter easily to transfer to the clinic as a diagnostic tool to study autophagy in disease or aging.

## 2 Materials and methods

### 2.1 Blood collection

#### 2.1.1 Mice

Male C57Bl/6J (B6; original strain Jackson Lab) and obese New Zealand Obese (NZO; NZO/HIBomDife mice, German Institute of Human Nutrition, Potsdam-Rehbruecke, Germany) mice were obtained from in-house breeding and housed under 12 h light/dark cycle with water and food *ad libitum*. All mice received a rodent standard diet (V1534, ssniff Spezialdiäten GmbH, Soest, Germany). For blood collection, mice were anesthetized and blood was collected by puncture of the heart or vena cava using EDTA rinsed cannula. Housing conditions and animal experiments were carried out according to German law on protection of animals. No further ethical approval was necessary because mice were only sacrificed to collect organs and tissue (§4 Abs. 3 TierschG).

#### 2.1.2 Human

Human samples were collected from non-diabetic women in term of the ChronoFast study registered under NCT04351672 (17 April 2020) at ClinicalTrials.gov. The study was invented by the research group Molecular Nutritional Medicine of the German Institute of Human Nutrition. All participants gave written informed consent. The study protocol was approved by the ethics committee of the University of Potsdam and was in accordance with the declaration of Helsinki of 1975.

### 2.2 PBMC isolation

#### 2.2.1 Mice

PBMC isolation was done following a standard protocol (GE Healthcare, 2010). 600–1,000 µL blood were filled up with 0.14 M NaCl to increase the volume up to 4 mL. 3 mL Ficoll™-Paque Plus (*ρ* = 1.077 g/cm³; GE14-1440-03, Cytiva) were pipetted onto bottom of a 15 mL conical centrifuge tube. Diluted blood was carefully layered on top of the gradient and centrifuged at 400 x g for 40 min at 20°C, with brake off. After centrifugation, the top layer containing serum and NaCl and the PBMC layer (buffy coat) were carefully collected.

##### 2.2.1.1 Isolation of murine PBMCs using a density gradient media with 1.080 g/cm³

A 55% Percoll solution was prepared following Mizobe et al. (Mizobe et al., 1982). The density was checked with an areometer for *ρ* = 1.080 g/cm³. Cells were isolated from 20–23 weeks (w) old male B6 and NZO mice. Isolation was done as previously described.

#### 2.2.2 Human

Blood was collected in BD Vacutainer^®^ Mononuclear Cell Preparation Tubes (362782, BD Medical) containing density gradient Ficoll™ (*ρ* = 1.077 g/cm³) and anticoagulant Na_3_ Citrate. Tubes were stored for 20 min at RT to reduce temperature effect on isolation. Blood was centrifuged at 1,650 × g for 20 min at 20°C, with brake on. After centrifugation, tubes contain a stable barrier separating PBMCs and serum from other cell types. The vacutainer was turned upside down to mix PBMC and serum. Cell suspension was collected in 15 mL conical centrifuge tubes.

### 2.3 Cardiomyocyte isolation

Cardiomyocytes were isolated according to an adjusted protocol based on the Langendorff-Free Method from Ackers-Johnson et al. (2016). In short, the animal was anesthetized using isoflurane followed by cervical dislocation. The heart was removed and transferred into a dish containing EDTA solution. A special designed cannula with an indentation was used on a 1 mL syringe filled with EDTA solution. The aorta was placed on the cannula and fixed with a thread. 1 mL EDTA solution was released through the aorta directly into the heart. The heart fixed on the cannula was connected to a pump rinsing the heart with 10 mL EDTA solution followed by 3 mL perfusion buffer. Afterwards, the heart was digested with a collagenase buffer for 15 min at 37°C. Heart tissue was mechanically dissociated and stop solution was added. Cell suspension was passed through a 100 µm pore-size strainer. In 3 steps (each 10 min) cardiomyocytes were separated from non-myocytes by gravity settings and calcium was reintroduced. The pellet was washed with PBS and resuspended in plating medium. Myocytes were transferred to laminin-coated 6-Well cell culture plates and allowed to seed for 1 h at culturing conditions. Thereafter, plating medium was replaced by culture medium.

### 2.4 Treatment with ConA

In previous experiments, we compared inhibitor treatment using chloroquine (CQ) and ConA for 6 and 24 h in adult cardiomyocytes, demonstrating the comparable inhibitory effects of both compounds on LC3A/B-I, LC3A/B-II and SQSTM1 expression (unpublished data). From our results, both inhibitors act quite similar, even if ConA seems to be more efficient in short-time treatment, as it was able to increase LC3A/B-II:LC3A/B-I ratio already after 6 h contrary to CQ. Thus, we decided to use ConA instead of CQ as we aimed to use a short-time inhibition of 2 h in our samples and additionally verified the inhibitory effect by measurement of lysosomal activity.

#### 2.4.1 PBMCs

Collected PBMC suspension was split into two 15 mL conical centrifuge tubes. ConA was used at a final concentration of 100 nM to minimize possible effects of solvent DMSO. ConA (C 9705, Sigma) was dissolved in DMSO (A994.2, Roth) for a stock solution of 1 mM and further diluted to 20 µM in 0.14 M NaCl. Finally, pre-dilution was applied 1:200 to obtain the final concentration of 100 nM ConA. Final DMSO concentration for control and ConA samples was 0.01%. Samples were incubated with ConA or DMSO at 37°C for 2 h. Afterwards, cells were washed twice in 10 mL phosphate-buffered saline (PBS; 137 mM NaCl, 2.7 mM KCl, 1.5 mM KH_2_PO_4_, 8.1 mM Na_2_HPO_4_*2H_2_O; pH = 7.4), centrifuged at 400 × g, 10 min. 4°C and 100 × g for 10 min at 4°C. Then, pelleted PBMCs were resuspended in 1 mL PBS and transferred to a 1.5 mL microcentrifuge tube. PBMCs were centrifuged at 400 × g for 5 min at 4°C. Supernatant was discarded and pellet was stored at −80°.

#### 2.4.2 Cardiomyocytes

1 mM ConA stock solution was diluted in culture medium to obtain a final concentration of 5 nM, determined by previous toxicity assay for the cardiomyocytes (data not shown). Final DMSO concentration for control and ConA samples was 0.00005%. Cells were incubated with ConA and DMSO for 2 h at 37°C, 5% CO_2_. Then, cardiomyocytes were washed twice with PBS containing 10 mM 2,3-Butandion-monoxim (B0753, Sigma-Aldrich) and scraped off the plate. After centrifugation, cell pellet was snap frozen in liquid nitrogen and stored at −80°C.

### 2.5 May Grunwald-Giemsa staining

To stain different blood cell types, 10 µL of the PBMCs suspension was pipetted onto a glass slide and left to dry. Cells were fixed for 5 min in methanol and washed two times in Sorensen buffer (0.102 M KH_2_PO_4_, 0.98 M K₂HO₄P * 3 H₂O, pH = 6.8). Fixed cells were stained with May Grunwald solution (63590, Sigma; 50% May Grunwald solution, 50% Sorensen buffer) for 6 min and incubated in Giemsa solution (48900, Sigma; 10% Giemsa solution, 90% Sorensen buffer) for 10 min. Afterwards, cells were washed twice with Sorensen buffer and water. On the dried slide, cell types were counted under a microscope and proportion of PBMCs in total cell count was calculated.

### 2.6 Lysosomal activity

Isolated PBMCs were dissolved in 100 (mice) or 120 µL (human) 1 mM DTT (6908.3, Roth) and shaken at 1,400 rpm for 1 h at 4°C. Lysates were sonicated at 80% amplitude for 10 s on ice (Dr Hilscher GmbH, Germany). Protein was measured by Bradford assay and 0.4–2.5 µg protein were mixed with 100 µL incubation buffer (150 mM Na-acetate, 24 mM cysteine * HCl, 3 mM EDTA dihydrate). Lysates were shaken for 2 min followed by an incubation for 10 min at RT. Fluorogenic peptide Z-Phe-Arg-AMC (BML-139, Enzo) was used as a substrate at 167 µM. Fluorescence released by splitting of the peptide from AMC was measured in a black 96-well plate at 380/460 nm every 3 min for 75 min (murine) or 120 min (human). Activity was calculated using free AMC standards (40113690025, Bachem).

### 2.7 Western blotting

Murine protein lysates obtained from lysosomal activity assay lysis were precipitated by acetone overnight at −20°C. Lysates were centrifugated at 14000 x g for 20 min at 4°C, acetone was discarded and pellet was left to dry. Human protein lysates were diluted in 1 mM DTT. Laemmli buffer [0.25 mM Tris (pH = 6.8), 40% Glycerol, 20% 2-Mercaptoethanol, 8% SDS, 0.03% Bromophenol Blue] was added to lysates and proteins were denatured for 5 min at 95°C. SDS polyacrylamide gel electrophoresis was used to separate 4–30 µg of the protein (same amount for control and ConA treated samples). Proteins were transferred onto 0.2 µm nitrocellulose membranes (10600001, Cytiva) and blocked in PBS containing 5% BSA (8076.3, Roth) and 0.01% sodium azide for 1 h at RT. Primary antibodies were diluted in blocking buffer adding 0.1% Tween^®^ 20 (P9416, Sigma-Aldrich) and incubated overnight at 4°C. Following antibodies were used: anti-GAPDH antibody (1:20,000, RRID: AB_2107448, ab8245, Abcam), anti-SQSTM1/p62 antibody (1:5,000, RRID: AB_945626, ab56416, Abcam), anti-LC3A/B antibody (1:2,000, RRID: AB_2617131, 1,274, Cell Signaling). Membranes were washed with PBS. Afterwards, fluorescent-labeled secondary antibodies (962-68022 and 925-32211, LI-COR), diluted in blocking buffer with 0.1% Tween^®^ 20, were added for 1 h at RT. Membranes were washed with PBS and scanned by Odyssey^®^ CLx Imaging System (LI-COR) and analyzed using Image Studio Software (v 5.2, LI-COR).

### 2.8 Immunofluorescence staining

50 µL of the PBMCs suspension were diluted with 150 µL PBS on a glass bottom dish (P35G-1.0.14-C, MatTek). After 30 min at RT, supernatant was removed and cells were fixed with 4% formaldehyde (LC-6470.5, Labochem) for 30 min at RT. Cells were washed with PBS and stored covered with PBS at 4°C until staining. PBMCs were permeabilized using 0.1% Triton™ X-100 (T8787, Sigma-Aldrich) in PBS for 15 min and washed with PBS. 1% FCS (F2442, Sigma) in PBS was used for 1 h blocking at RT. Primary antibody anti-TFEB (RRID: AB_2808434, PA5-96632, Thermo Fisher) was diluted 1:100 in blocking buffer and added for 2 h at RT. PBMCs were washed with PBS and incubated with the Alexa Fluor^®^ 488 labeled secondary antibody (A-11008, Thermo Fisher) diluted in blocking buffer for 1 h at RT. Cells were washed with PBS and one drop ROTI®Mount FluorCare DAPI (HP20.1, Roth) was added as mounting medium.

Visualization was performed at room temperature using a Zeiss LSM 780 confocal laser scanning microscope with objective Plan-Apochromat 63x/1.40 Oil DIC M27 (Zeiss, Germany) and the software provided with the microscope (Zen lite 3.0; Zeiss, Germany). Fluorescence signal of TFEB was determined at DAPI and non-DAPI expressing area of a cell. At least 33 cells per sample were analyzed. Evaluation was done using Fiji (v 1.53q).

### 2.9 Statistical analysis

Statistical analyses were performed using GraphPad Prism (v. 9.3.1). Shapiro Wilk test was used to verify normal distribution. In murine samples, ConA-induced changes compared to controls were given as x-fold change of induction, as untreated mice already showed high individual variations. Therefore, statistical differences were analyzed using one-sample Wilcoxon test. Differences between young and old murine samples and ConA-induced alterations in human PBMCs were determined using unpaired *t*-test or, if normality test failed, Mann-Whitney U test. Results are presented as mean values ± SD and statistical significance was considered at *p* ≤ 0.05 and given as follows: *p ≤ 0.05, **p ≤ 0.01, ***p ≤ 0.001, ****p ≤ 0.0001.

## 3 Results

### 3.1 Verification of the isolation method for murine PBMCs

**FIGURE 1:**
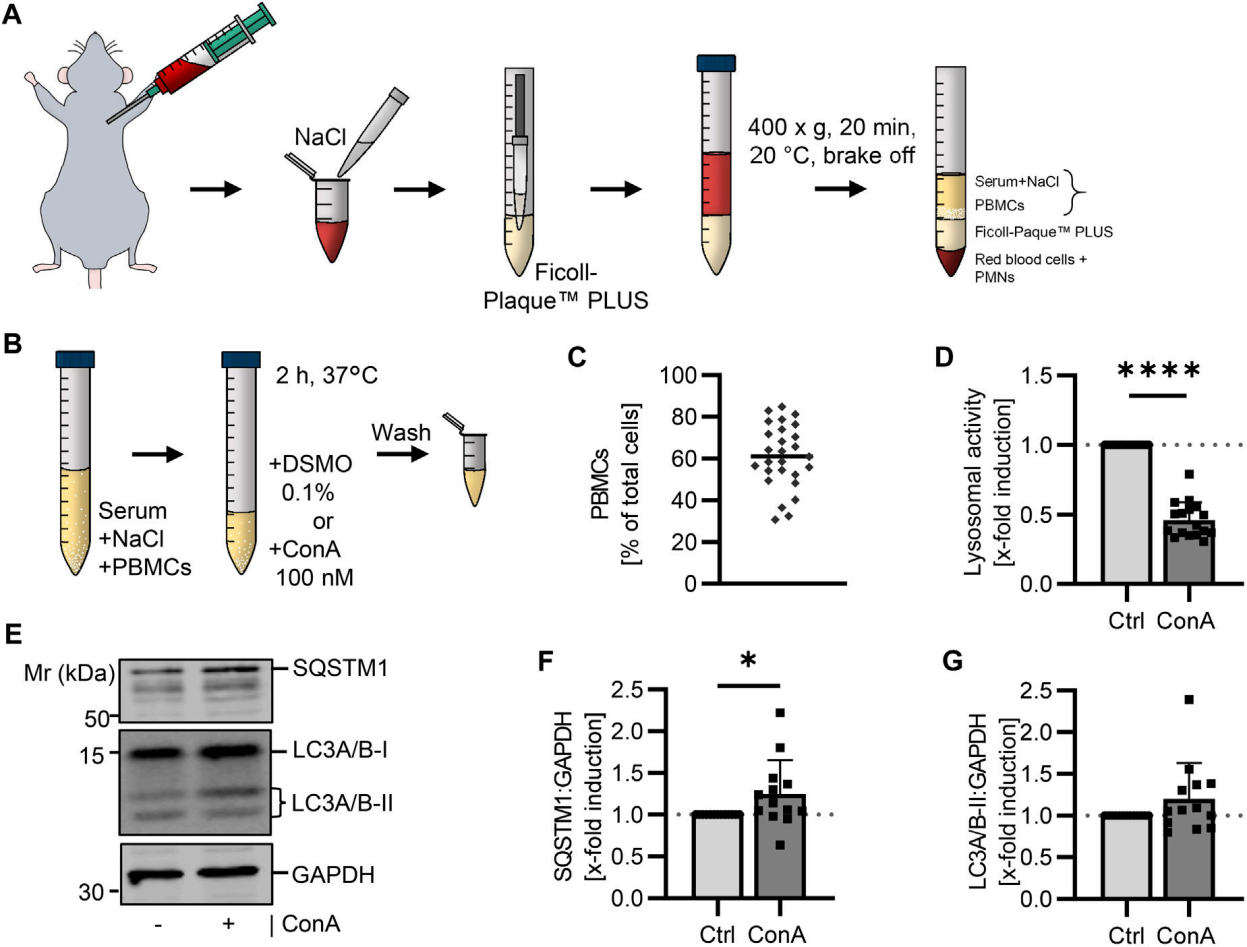
Determination of the autophagic flux in isolated murine PBMCs using a lysosomal inhibitor. **(A)** Blood was collected from young (21-22 w) B6 mice. The whole blood was diluted with NaCl and layered above Ficoll-Paque™ PLUS. After centrifugation, serum (diluted with NaCl) and PBMCs were carefully collected. **(B)** Murine PBMCs were treated in serum-NaCl solution with 100 nM ConA for 2 h at 37°C. **(C)** Percentage of PBMCs on total isolated cells was counted after May Grunwald-Giemsa stain (*n* = 27). **(D)** Lysosomal activity was decreased in ConA treated PBMCs. Activity was measured by Cathepsin B and L mediated release of AMC after 75 min (*n* = 16). **(E)** Representative western blot for SQSTM1, LC3A/B-I, LC3A/B-II and GAPDH in control and ConA treated PBMCs. **(F)** SQSTM1 normalized to GAPDH was enhanced in ConA treated PBMCs (*n* = 13). **(G)** LC3A/B-II normalized to GAPDH was not affected by ConA treatment. Data represent mean ± SD. Statistical significance was tested with one sample Wilcoxon test and given as follows: *p ≤ 0.05, **p ≤ 0.01, ****p ≤ 0.0001. Mr, molecular weight marker.

Whole murine blood was diluted with 0.14 M NaCl and PBMCs were isolated by density gradient centrifugation using Ficoll™-Paque PLUS with 1.077 g/cm³. Fraction of PBMCs, serum and NaCl solution was collected for ConA treatment (Figure 1A). Toxicity of ConA was tested by trypan blue staining and showed no effect at the used concentration (Table S1). This allowed us to be as near as possible to physiological conditions instead of culturing the cells in complete medium. After treating the PBMCs with ConA for 2 h at 37°C, cells were washed and prepared for further experiments (Figure 1B). Isolations were performed at different days to verify reproducibility. Isolated cells were stained with May Grunwald-Giemsa staining to determine the distribution of different blood cell types. The percentage of PBMCs on whole isolated cell fraction was on average 61.1% (Figure 1C).

### 3.2 Effect of density gradient media on murine PBMC isolation

In the literature it is described, that PBMCs from rodents have a slightly higher density than PBMCs from humans (Mizobe et al., 1982; Bøyum et al., 1991; GE Healthcare, 2010). Therefore, we additionally tested a 55% Percoll solution with 1.080 g/cm³ for PBMC isolation. After centrifugation, a red layer was present between buffy coat and density gradient medium, which we did not observe after centrifugation with Ficoll™-Paque PLUS (Supplementary Figure S1A, B). Moreover, a proportion of 43.9% ± 4.7% PBMCs in the isolated fraction from B6 mice using Percoll was determined. To check for another mouse strain, blood from NZO mice, an obese mouse model, suffering from high serum triglycerides and heart failure (Ott et al., 2021), was used. Here, isolated cells consisted of 73.3% ± 13.7% PBMCs using the same 55% Percoll solution (Supplementary Figure S1C).

### 3.3 Lysosomal activity decreases while SQSTM1 protein level increases in ConA treated murine PBMCs

To verify the inhibiting effect of ConA on lysosomal activity, maximal lysosomal-associated cathepsin activity was measured *via* enzyme activity assay. To analyze the autophagic flux, SQSTM1 and LC3A/B protein levels in control and ConA-treated samples were determined by western blot and normalized to Glyceraldehyde-3-phosphate dehydrogenase (GAPDH). Activity of lysosomal cathepsins was decreased by ConA, remaining activity of 44.2% ± 9.8% (Figure 1D). SQSTM1 protein levels were enhanced after 2 h ConA treatment (Figures 1E, F). LC3A/B-II was not affected by ConA treatment (*p* = 0.16; Figures 1E, G). However, LC3A/B-II:LC3A/B-I ratio (both normalized to GAPDH) was increased, while LC3A/B-I form was unchanged by ConA (*p* = 0.005 for LC3A/B-II:LC3A/B-I vs. *p* = 0.58 for LC3A/B-I; Supplementary Figures S2A, B).

### 3.4 TFEB is not altered by lysosomal inhibition

**FIGURE 2:**
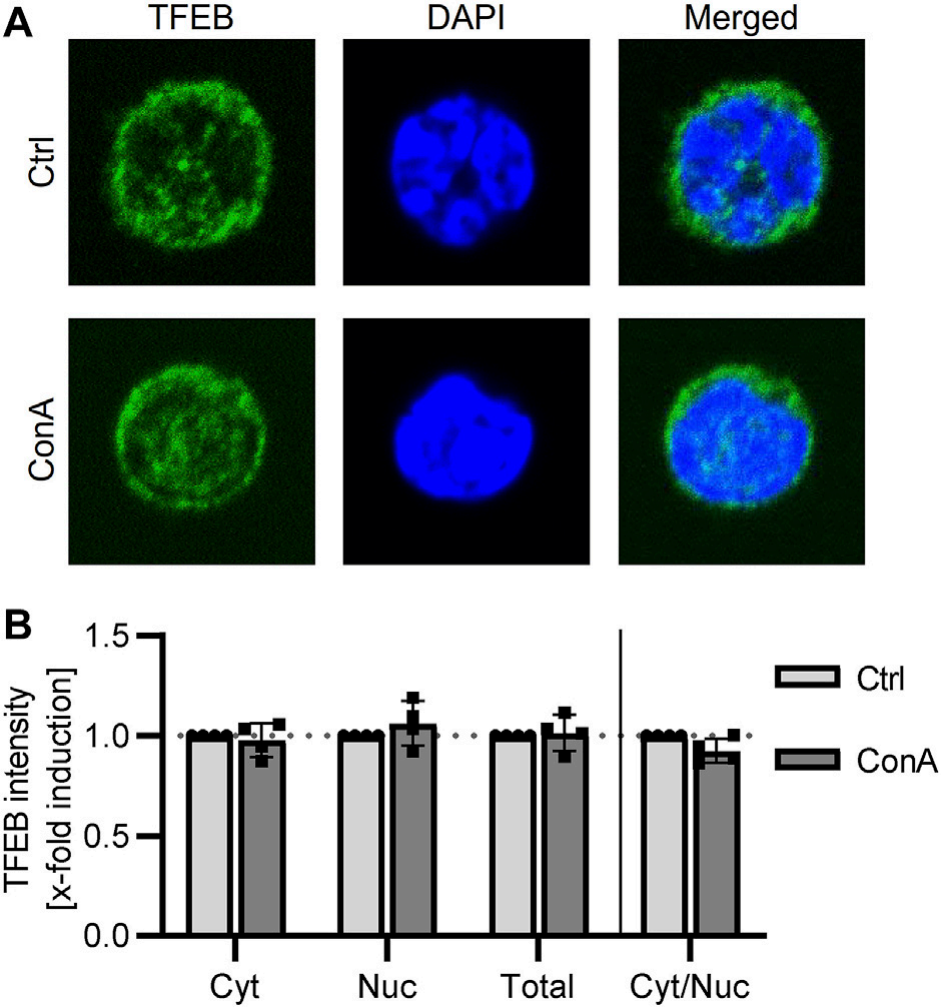
TFEB location is not altered by ConA treatment in murine PBMCs. PBMCs from young mice were treated for 2 h with 100 nM of ConA at 37°C. **(A)** Representative immunofluorescent staining for TFEB (green), DAPI (blue) and merged channels in Ctrl and ConA treated PBMCs. **(B)** ConA treatment did not change cytosolic, nuclear, total TFEB intensity or the ratio of cytosolic to nuclear TFEB signal of murine PBMCs (*n* = 4). Data represent mean ± SD. Statistical significance was tested with one sample Wilcoxon test.

Translocation of TFEB into the nucleus initiates expression of autophagic and lysosomal proteins (Napolitano and Ballabio, 2016). Therefore, we stained TFEB and analyzed its signal in cytosol, nucleus and in total (cytosol and nucleus) in PBMCs treated with or without ConA for 2 h. Comparing control and ConA treated PBMCs, cytosolic, nuclear and total TFEB intensity and ratio of cytosolic to nuclear TFEB were not significant altered yet (Figure 2).

### 3.5 Aging affects the autophagic flux in murine PBMCs

**FIGURE 3:**
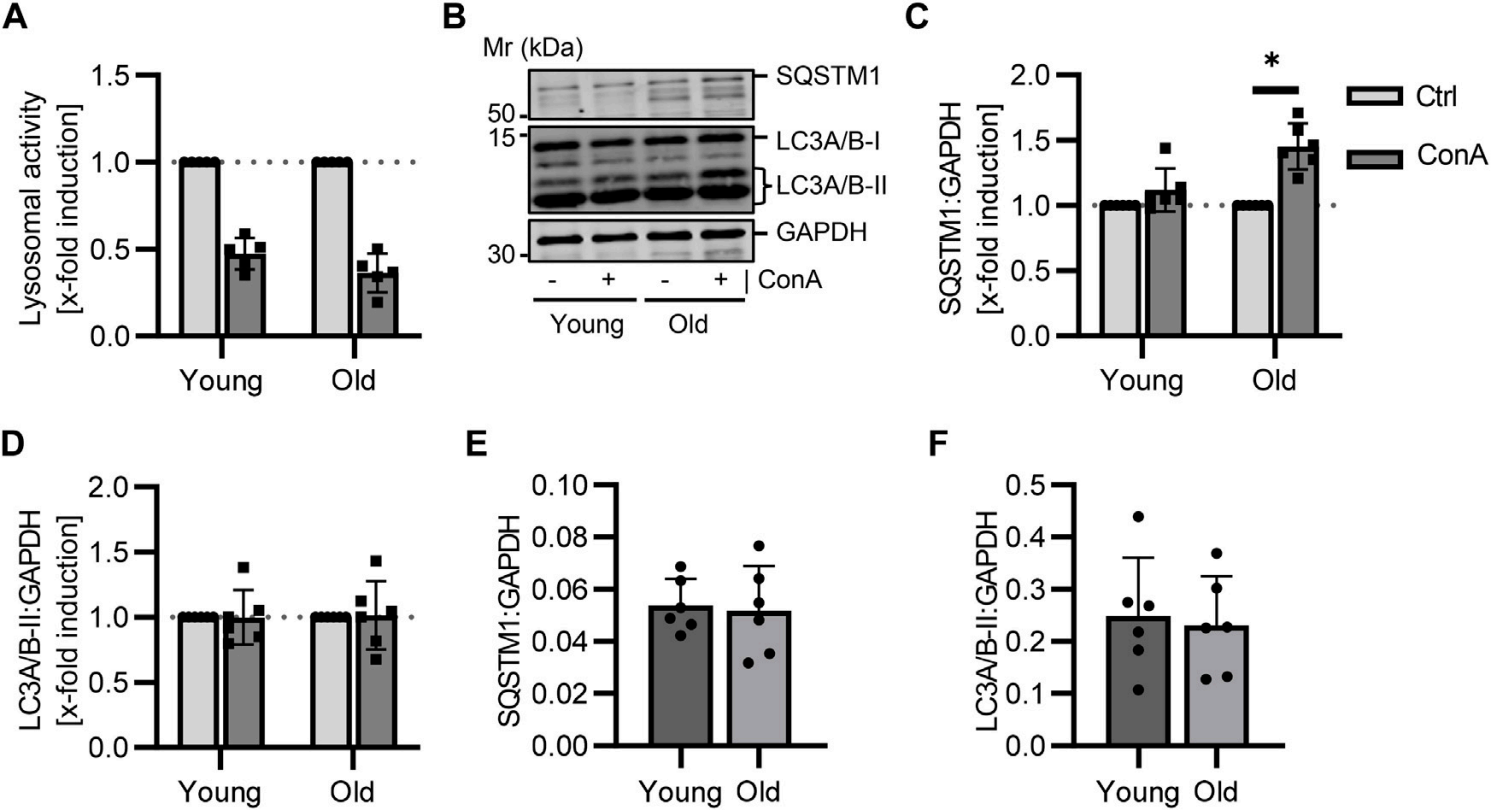
Aging affects the autophagy receptor SQSTM1 in murine PBMCs. Isolated PBMCs from young and old mice were treated with ConA for 2 h at 37°C (*n* = 5-6 per age). **(A)** Inhibition of lysosomal activity was not altered by age in murine PBMCs **(B)** Representative western blot for SQSTM1, LC3A/B-I, LC3A/B-II and GAPDH in control and ConA treated PBMCs from young and old mice. **(C)** SQSTM1 to GAPDH turnover was increased in old PBMCs due to ConA treatment, **(D)** while LC3A/B-II to GAPDH was not affected. **(E)** Control SQSTM1 and **(F)** LC3A/B-II protein levels murine PBMCs showed no aging effect. Data represent mean ± SD. Statistical significance was tested with one sample Wilcoxon test **(A–D)** or *t*-test **(E, F)** and given as follows: *p ≤ 0.05. Mr, molecular weight marker.

PBMCs were isolated from young (21-22 w) and old (105-106 w) B6 mice followed by 2 h ConA treatment. The aim was to measure, whether the presented method is able to show changes on autophagy that occur by age. A trend to reduced lysosomal activity was measured in PBMCs of both age groups (Figure 3A; *p* = 0.06 for both). SQSTM1 protein levels were enhanced in ConA-treated PBMCs from old but not in PBMCs from young mice (Figures 3B, C; *p* = 0.03 vs. *p* = 0.06). LC3A/B-II was not significantly affected by ConA in both age groups (Figures 3B, D). Also, values of LC3A/B-I and the LC3A/B-II:LC3A/B-I ratio were not altered yet (Supplementary Figures S3A, B). Comparison of control samples from both groups showed no age effect on lysosomal activity, the expression of autophagy receptor SQSTM1 and both LC3A/B forms (Figures 3E, F; Supplementary Figures S3C, D). Although, basal LC3A/B lipidation was slightly lower in PBMCs from old mice compared to young mice (Supplementary Figure S3E; *p* = 0.07).

### 3.6 PBMCs show different autophagic flux compared to isolated cardiomyocytes

**FIGURE 4:**
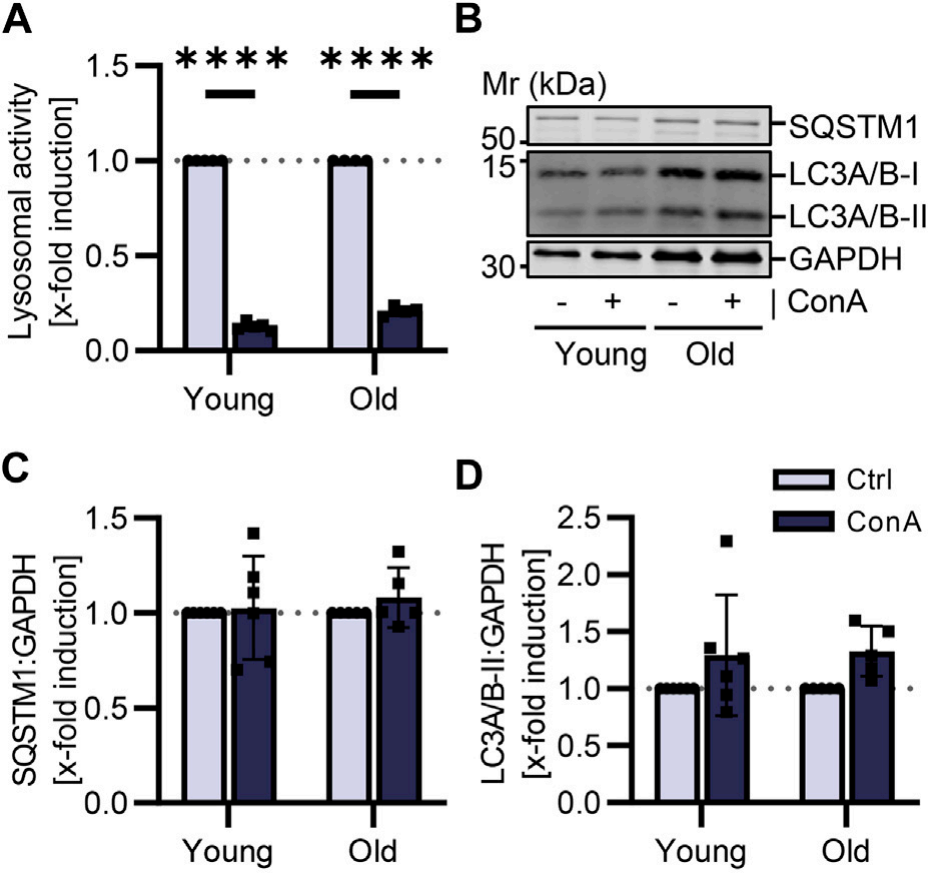
Autophagic flux was not affected by aging in murine cardiomyocytes. Cardiomyocytes were isolated from young (21-22 w) and old (105-106 w) mice and treated for 2 h at 37°C with 100 nM ConA. **(A)** Lysosomal activity of murine cardiomyocytes was decreased by ConA in both age groups (*n* = 4–5). **(B)** Representative western blot for SQSTM1, LC3A/B-I, LC3A/B-II and GAPDH in control and ConA treated cardiomyocytes from young and old mice. **(C)** In young and old cardiomyocytes, SQSTM1 and **(D)** LC3A/B-II both normalized to GAPDH were not changed by ConA treatment (n = 5–6). Data represent mean ± SD. Statistical significance was tested with one sample Wilcoxon test and given as follows: ****p ≤ 0.0001. Mr, molecular weight marker.

Cardiomyocytes were isolated from the same young and old mice as PBMCs. To compare data on the autophagic flux and to determine how conclusive results from blood cells are for other cell types, primary adult cardiomyocytes were also incubated with lysosomal inhibitor ConA for 2 h. Lysosomal activity was significantly reduced by ConA treatment in cardiomyocytes from both age groups (Figure 4A). However, ConA did not affect protein expression of SQSTM1 in cardiomyocytes (Figures 4B, C). LC3A/B-II was slightly enhanced in old cardiomyocytes due to ConA treatment (Figures 4B, D; *p* = 0.22 vs. *p* = 0.06).

### 3.7 Autophagic flux can also be measured in human PBMCs

**FIGURE 5:**
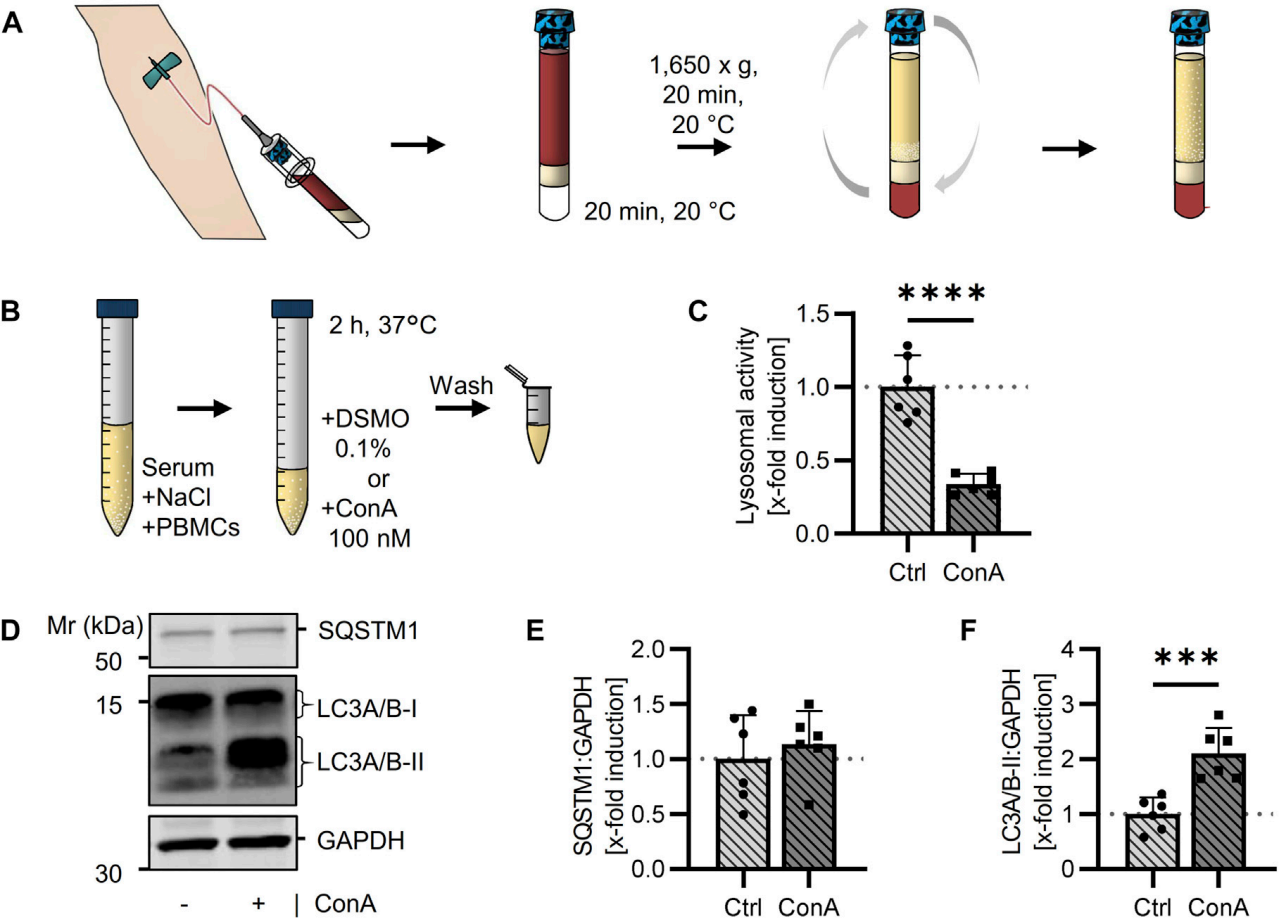
Measurement of the autophagic flux in isolated human PBMCs using a lysosomal inhibitor. **(A)** Human blood was taken directly into BD Vacutainer^®^ with CPT™ system followed by a centrifugation. PBMCs were dissolved in serum by turning the tube upside down. **(B)** Collected PBMC cell suspension was split into 2 samples, either treated with DMSO (Ctrl) or 100 nM ConA for 2 h at 37°C. After washing, cells were extracted for following experiments. **(C)** Lysosomal activity was decreased in ConA treated PBMCs. Activity was measured by Cathepsin B and L mediated release of AMC after 90 min (*n* = 6). **(D)** Representative western blot for SQSTM1, LC3A/B-I, LC3A/B-II and GAPDH in control and ConA treated PBMCs. **(E)** SQSTM1 normalized to GAPDH was not altered by ConA treatment (*n* = 6). **(F)** LC3A/B-II to GAPDH was increased in PBMCs treated with ConA compared to control cells. Data represent mean ± SD. Statistical significance was tested with *t*-test and given as follows: ***p ≤ 0.001, ****p ≤ 0.0001. Mr, molecular weight marker.

Human PBMCs were isolated from whole blood using BD Vacutainer^®^ with CPT system (Figure 5A). These vacutainers simplify the isolation because blood dilution or pipetting of the density gradient medium is not required. Moreover, after centrifugation PBMCs and serum are permanently separated from other blood cells and PBMCs can easily be resolved in their serum by upending the vacutainers (Schlenke et al., 1998). Resolved PBMCs were treated in their serum with ConA as done in murine PBMCs (Figure 5B). Toxicity of ConA was tested by trypan blue staining and showed no effect at the used concertation (Table S1). Experiments were performed at different days and with blood from different human subjects to verify reproducibility. In human PBMCs, ConA treatment resulted in decreased lysosomal activity of 33.8% ± 7.1% (Figure 5C). SQSTM1 protein levels were not altered by ConA treatment (Figures 5D, E). LC3A/B-II expression was strongly upregulated by ConA, while LC3A/B-I level remained unaffected (Figures 5D, F), suggesting an enhanced LC3A/B-II accumulation mediated by ConA in human PBMCs (Supplementary Figures S4A, B).

## 4 Discussion

In this study, we evaluate an easy-to-use method for measuring the autophagic flux in murine and human isolated PBMCs. To our knowledge, this is the first time a method is describing determination of autophagic flux in murine PBMCs, additionally usable for human samples. Our protocols allow autophagic flux investigation of PBMCs in their blood serum directly within 2 h and without culturing cells in complete medium, being as near to physiological conditions as possible.

To create a protocol for the determination of autophagy in murine PBMCs, first aim was to establish the density gradient isolation of PBMCs from murine blood as protocols are limited. Initially, isolation was tested with two different density gradient media, as these are still discussed in the literature. For instance, GE Healthcare recommended a density gradient of 1.084 g/cm³ for isolation of rodent PBMCs, which is slightly higher than the density gradient used for human PBMC isolation (1.077 g/cm³) (GE Healthcare, 2010). However, Mizobe et al. were not able to determine the indicated differences in densities of murine and human PBMCs (Mizobe et al., 1982). Regarding the literature different density gradients were applied for PBMC isolation. For instance, PBMC isolation was published using 1.077 g/cm³ in C57BL/6 mice (Mendez-David et al., 2013; Song et al., 2021), whereas 1.084 g/cm³ was used for MRL(-Ipr) mice (Wang et al., 2018). Based on the literature, we tried for murine PBMC isolation a Percoll solution with 1.080 g/cm³ (Mizobe et al., 1982) and compared this to Ficoll™-Plaque PLUS with 1.077 g/cm³. PBMC isolation using Percoll resulted in a higher contamination of the isolated cell fraction compared to Ficoll™-Plaque PLUS (56% vs. 39%; Figure 1C; Supplementary Figure S1C). As shown in Figure 1C mean percentage of isolated PBMCs from murine blood samples was 61%. To our knowledge, comparable data on murine PBMC yields are still not sufficiently known. For PBMCs isolated from human blood (using BD Vacutainer^®^ with CPT™ system), manufacturers provide contamination yields of 16%, leading to an average of 84% PBMCs on the whole isolated cell fraction after centrifugation. To investigate whether density gradient can also be used for the isolation of PBMCs from another mouse strain, blood collected from NZO mice was used. The NZO strain is used as an obesity model. On standard diet, mice already develop a metabolic phenotype described by obesity and high triglyceride levels with additional left ventricular failure (Ott et al., 2021), whereas mice on a certain high-fat high carb diet regime show a type 2 diabetes-like syndrome (Ortlepp et al., 2000). In humans, type 2 diabetes mellitus was associated with more red blood cells of high density compared to healthy controls (Bizjak et al., 2015). This could explain lower red blood cell contamination in PBMCs isolated from NZO mice compared to B6 mice (Supplementary Figure S1), as more erythrocytes were able to migrate through the density gradient media. Therefore, in these mice Percoll led to a higher yield of PBMCs than Ficoll™-Plaque PLUS (Supplementary Figure S1), even if mice received the same standard diet. However, blood glucose and distribution of red blood cells were not further investigated in this study.

After isolation of PBMCs, cells were directly treated with lysosomal inhibitor ConA in their serum, to determine autophagic flux. In murine samples, filling up of serum with NaCl was necessary to increase the volume needed for isolation, due to the low amount of total blood volume obtained from mice. Inhibitor ConA was used to increase lysosomal pH by blocking lysosomal V-ATPases (Huss et al., 2002), verified by a strong decrease in lysosomal-associated cathepsins activity (Figure 1D).

To proof ConA-related autophagic flux manipulation, SQSTM1 and LC3A/B were analyzed. SQSTM1 works as a receptor for autophagic cargo but also gets degraded in autolysosomes. Its accumulation is used as an index for autophagic degradation (Klionsky et al., 2021b). In murine PBMCs, ConA treatment resulted in enhanced SQSTM1 protein levels compared to control (Figure 1F). Quantification of SQSTM1 is often used in combination with the determination of lipidated LC3 as LC3-II correlates with number of autophagosomes (Mizushima and Yoshimori, 2007). However, LC3A/B-II was not significantly increased in ConA treated murine PBMCs (Figure 1G). While LC3A/B-I was also not altered, the ratio of LC3A/B-II:LC3A/B-I in presence of the inhibitor was significantly increased, compared to the non-treated control, indicating the 2 h inhibition of lysosomal activity led to an increased formation of LC3A/B-II from LC3A/B-I (Supplementary Figure S2).

To determine, if lysosomal inhibition results in an induction of autophagic and lysosomal associated genes, cellular location of TFEB was analyzed by microscopy. TFEB translocation into the nucleus induces transcription of genes from the CLEAR network (Sardiello et al., 2009). In our experiments, ConA treatment was not affecting nuclear, cytosolic or TFEB total stained area. Additionally, cytosolic to nuclear ratio of TFEB signal was not affected yet (Figure 2). A recommended short incubation time of 2 h was used in this study to limit *ex vivo* effects on cells (Klionsky et al., 2021b). Nevertheless, longer incubation times (>8 h) were related to TFEB translocation (Roczniak-Ferguson et al., 2012; Settembre et al., 2012). Atg5, required for LC3 lipidation, and SQSTM1 are TFEB target genes (Palmieri et al., 2011; Di Malta et al., 2019). The short incubation time as well as the small sample size could be reasonable for limited effects of ConA on TFEB translocation and in consequence on autophagic flux proteins.

Impact of aging on autophagic flux in murine PBMCs was assessed to investigate if age-related changes *in vivo* could be displayed by our method *ex vivo*. Loss of proteostasis is one hallmark of aging (López-Otín et al., 2013) and a decline in autophagy during aging has been observed in T and B lymphocytes (Zhou et al., 2021). In murine PBMCs, ConA induced SQSTM1 levels were measured in old but not in young PBMCs, while basal SQSTM1 and LC3A/B-II protein expression were not affected by aging (Figures 3C–F). Bresciani et al. (2018) showed that accumulation of SQSTM1 and LC3-II also rely on proliferation rate of the analyzed cell type, indicating slower proliferating cells show higher accumulation. During aging amount of senescent immune cells increased (Frasca et al., 2019), thereby possibly resulting in a stronger ConA effect because of their diminished proliferation rate. However, we could only verify this effect for SQSTM1, not LC3A/B-II protein levels, in murine PBMCs.

Contrary to our results, Mejías-Peña et al. reported lower basal LC3-II protein levels in old compared to young human subjects (Mejías-Peña et al., 2016). McCormick et al. measured a higher elevation in LC3-II protein levels by the lysosomal inhibitor in PBMCs from old than in PBMCs from young subjects, while SQSTM1 induction did not differ between the age groups. However, in their study PBMCs were cultured in complete medium for 24 h after isolation followed by 2 h bafilomycin treatment (McCormick et al., 2018).

To investigate whether results of the autophagic flux in PBMCs can be used as marker for autophagy status in other tissues, cardiomyocytes were utilized as another cell type. Cardiomyocytes were isolated from the same young and old mice as PBMCs. Isolation allowed us to not just measure basal expression of autophagic markers but to also investigate the autophagic flux by treating cardiomyocytes with lysosomal inhibitor ConA. Impact of aging on autophagic flux in PBMCs could not be reproduced in cardiomyocytes. We were not able to show any age differences in basal expression of autophagic markers or in the autophagic flux (Figure 4). Contrary to PBMCs, cardiomyocytes are postmitotic cells (Mohamed et al., 2022). These cells were treated with ConA for 2 h in a medium containing nutrients and hormones (Ackers-Johnson et al., 2016), that could impact autophagic flux. To our knowledge, this is the first time the autophagic flux was measured in isolated cardiomyocytes from young and old mice. Only Häseli et al. were able to determine increasing SQSTM1 protein levels in a senescence cardiomyocyte cell model using neonatal cells (Häseli et al., 2020). Often autophagy marker proteins were determined in heart tissue from young and old mice, however, results varied (Linton et al., 2015).

Besides murine samples, autophagic flux was also analyzed in PBMCs isolated from human blood samples (Figure 5), resulting in decreased lysosomal activity and increased LC3A/B-II protein levels induced by ConA. Our method is comparable to Bensalem et al., who evaluated a reproducible method for determination of the autophagic flux in human blood. In contrast to our method, the authors treated whole blood samples with the inhibitor chloroquine for 1 h, thereafter PBMCs were isolated using density gradient centrifugation. Chloroquine induced changes in LC3B-II protein expression were measured as an indicator for the autophagic flux. They determined an increase in SQSTM1 protein expression induced by chloroquine in 3 out of 4 subjects. Chloroquine treatment enhanced LC3B-II protein levels with increasing incubation time (Bensalem et al., 2021).

### 4.1 Advantages and limitations

The described method allows investigation of the autophagic flux in murine and human blood samples. A strong advantage is the possibility to treat PBMCs in their serum without cultivation in complete medium. Often PBMCs were cultured in Roswell Park Memorial Institute 1,640 medium followed by treatment with a lysosomal inhibitor (François et al., 2015; McCormick et al., 2018; Alsaleh et al., 2020). Although, culture medium contains nutrients and hormones, which could affect the autophagic flux (RostamiRad et al., 2018; Bensalem et al., 2021). Theoretically, these substance-related effects can be prevented with our method, however, we did not test or compare them in the presented study.

For human blood samples, PBMC isolation was easy to perform due to BD Vacutainer^®^ with CPT system, as the manually layering of the blood above the density gradient media is not necessary using these tubes. However, measuring the autophagic flux with this method is less suitable for large-scale in clinical studies. Cells have to be treated immediately with ConA after isolation to remain close to physiological conditions. The isolation of murine PBMCs has to be performed as fast as possible after blood collection to avoid hemolysis. Moreover, the amount of material in mice is limited due to the low amount of blood volume that you receive (600–1,000 µL).

To reduce the *ex vivo* effects on the autophagic flux, ConA incubation time was set to 2 h. However, short incubation time can also be reasonable for the minor changes in SQSTM1, LC3A/B-II and TFEB translocation. Besides short incubation time, sample size has to be considered due to high interindividual variations in both species. As nutritional status is one of the major autophagy modulators (Dikic and Elazar, 2018), high variation in mice and human could also be influenced by last food intake. Another aspect to point out is the possible diversity in cell types of the isolated PBMC fraction, as cell type composition can be different between samples from the same groups and therefore lead to interindividual variations in autophagy flux inhibition (Yousefi and Simon, 2009; Karagiannis et al., 2022).

Besides energy level, PBMC autophagy can be affected by infection and inflammation (Deretic, 2021). In hematopoietic cells, maintenance of autophagy plays an essential role, as hematopoietic cell specific deficiency of Atg7 in mice led to death within weeks (Mortensen et al., 2011). Furthermore, autophagy is necessary for B cell differentiation, T cell survival and in monocytes the mechanism is required for degradation of pathogens (Yousefi and Simon, 2009). In PBMCs from patients with chronic hepatitis B virus infection, expression of 18 autophagy-modulating genes was downregulated including GABARAPL1, Atg7 and CTSB as well as LC3-II protein levels (Tian et al., 2019). In addition, mRNA expressions of inflammatory markers IL-1β and TNF-α were negatively correlated with protein levels of LC3B-II and positively correlated with SQSTM1 in PBMCs from type 2 diabetes mellitus patients (Alizadeh et al., 2018). To exclude differences in infection or inflammation status, which also could justify interindividual discrepancies among the autophagy proteins, future studies should include measurement of inflammatory or oxidative stress marker.

In summary, we provide a new method to determine the autophagic flux in isolated murine and human PBMCs in presence and absence of a lysosomal inhibitor without completely changing environmental conditions. Future studies should focus on association between the autophagic flux in PBMCs and other tissues to determine how conclusive these results are for using PBMCs as marker for the autophagy status in different tissues. Effects of aging, obesity, inflammation and infection on autophagy status can be studied with this method in murine and human samples and can help monitoring autophagy-related diseases such as cancer, Alzheimer or Parkinson disease.

## Data Availability

The raw data supporting the conclusion of this article will be made available by the authors, without undue reservation.

## Abbreviations

ALS, autophagy lysosomal system; B6, C57BL/6J; ConA, concanamycin A; LC3, Microtubule-associated proteins 1A/1B light chain 3; NZO, New Zealand Obese; PBMCs, Peripheral blood mononuclear cells; PBS, phosphate-buffered saline; SQSTM1, Sequestosome 1; TFEB, Transcription factor EB; w, weeks.
