# Supplementary material for: Determination of the autophagic flux in murine and human peripheral blood mononuclear cells
Source: Front Cell Dev Biol. 2023 Mar 13;11:1122998. doi: 10.3389/fcell.2023.1122998 (PMC10040559; doi:10.3389/fcell.2023.1122998)
Supplement: Supplementary file 1 [file DataSheet1.docx]

Supplementary Material

**Supplementary Table 1:** Toxicity of concanamycin A treatment by trypan blue staining in PBMCs.

| **Species** | **Strain** | **Sample** | **Living cells** | **Dead cells (Stained)** | **Total cells** | **Living cells [%]** | **Mean** | **SD** |
| --- | --- | --- | --- | --- | --- | --- | --- | --- |
|  |  |  |  |  |  |  |  |  |
| **Mouse** | **C57BL6/J** | V | 139 | 6 | 145 | 95,862 | 95,571 | 0,971 |
|  |  |  | 159 | 6 | 165 | 96,364 |  |  |
|  |  |  | 120 | 7 | 127 | 94,488 |  |  |
|  |  | ConA | 150 | 7 | 157 | 95,541 | 96,015 | 0,692 |
|  |  |  | 182 | 6 | 188 | 96,809 |  |  |
|  |  |  | 200 | 9 | 209 | 95,694 |  |  |
|  | **NZO** | V | 232 | 4 | 236 | 98,305 | 96,494 | 1,667 |
|  |  |  | 150 | 6 | 156 | 96,154 |  |  |
|  |  |  | 210 | 11 | 221 | 95,023 |  |  |
|  |  | ConA | 264 | 9 | 273 | 96,703 | 97,478 | 0,718 |
|  |  |  | 209 | 4 | 213 | 98,122 |  |  |
|  |  |  | 204 | 5 | 209 | 97,608 |  |  |
| **Human** |  | V | 162 | 13 | 175 | 92,571 | 93,372 | 0,693 |
|  |  |  | 165 | 11 | 176 | 93,750 |  |  |
|  |  |  | 136 | 9 | 145 | 93,793 |  |  |
|  |  | ConA | 165 | 12 | 177 | 93,220 | 93,160 | 0,119 |
|  |  |  | 160 | 12 | 172 | 93,023 |  |  |
|  |  |  | 193 | 14 | 207 | 93,237 |  |  |


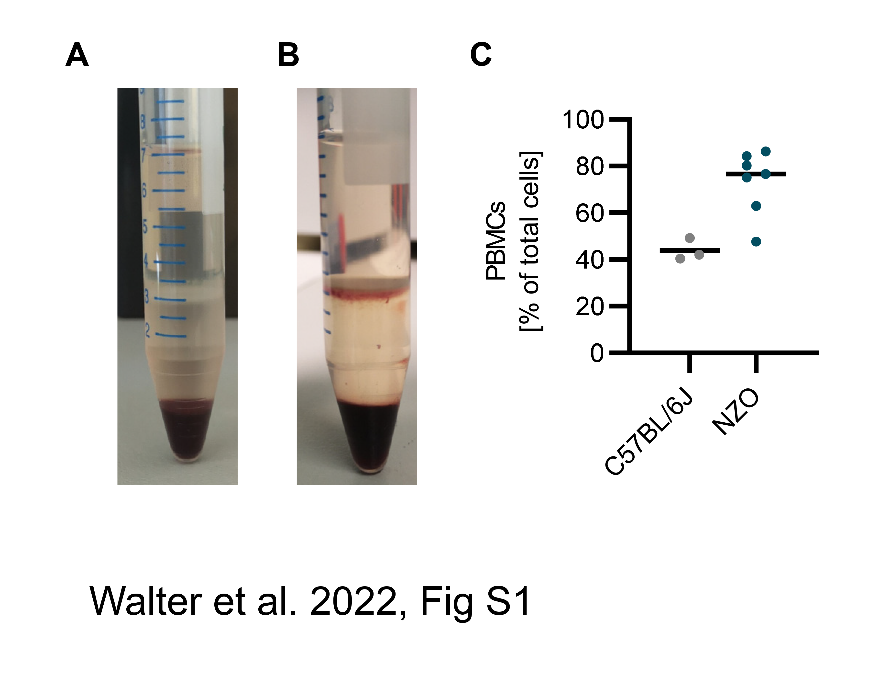


**Supplementary Figure 1:** Density gradient media impacts PBMC isolation from mice with different strains. Representative picture of PBMCs from B6 mice isolated with (A) Ficoll™-Plaque PLUS with 1.077 g/cm³ or (B) a 55% Percoll solution with 1.080 g/cm³. (C) PBMCs from B6 (n=3) and NZO mice (n=7) were isolated using Percoll with 1.080 g/cm³. Percentage of PBMCs on total isolated cell fraction was determined after May Grunwald-Giemsa staining.


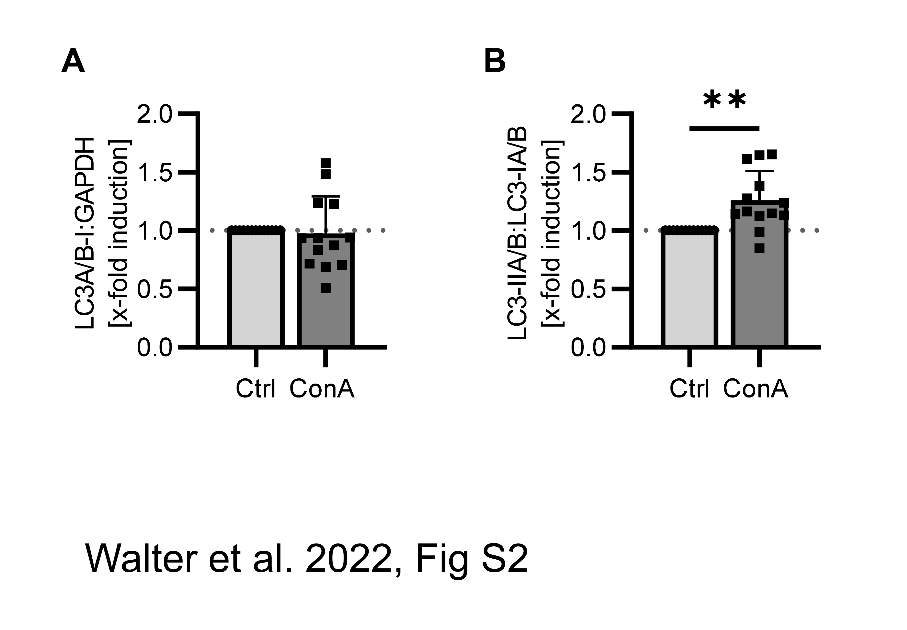


**Supplementary Figure 2:** LC3A/B lipidation is affected by 2 h ConA treatment. (A) LC3A/B-I and (B) LC3A/B-II:LC3A/B-I ratio protein levels were determined by western blot and normalized to GAPDH from control and ConA treated murine PBMCs (n=13). Data represent mean ± SD. Statistical significance was tested with one sample Wilcoxon test.


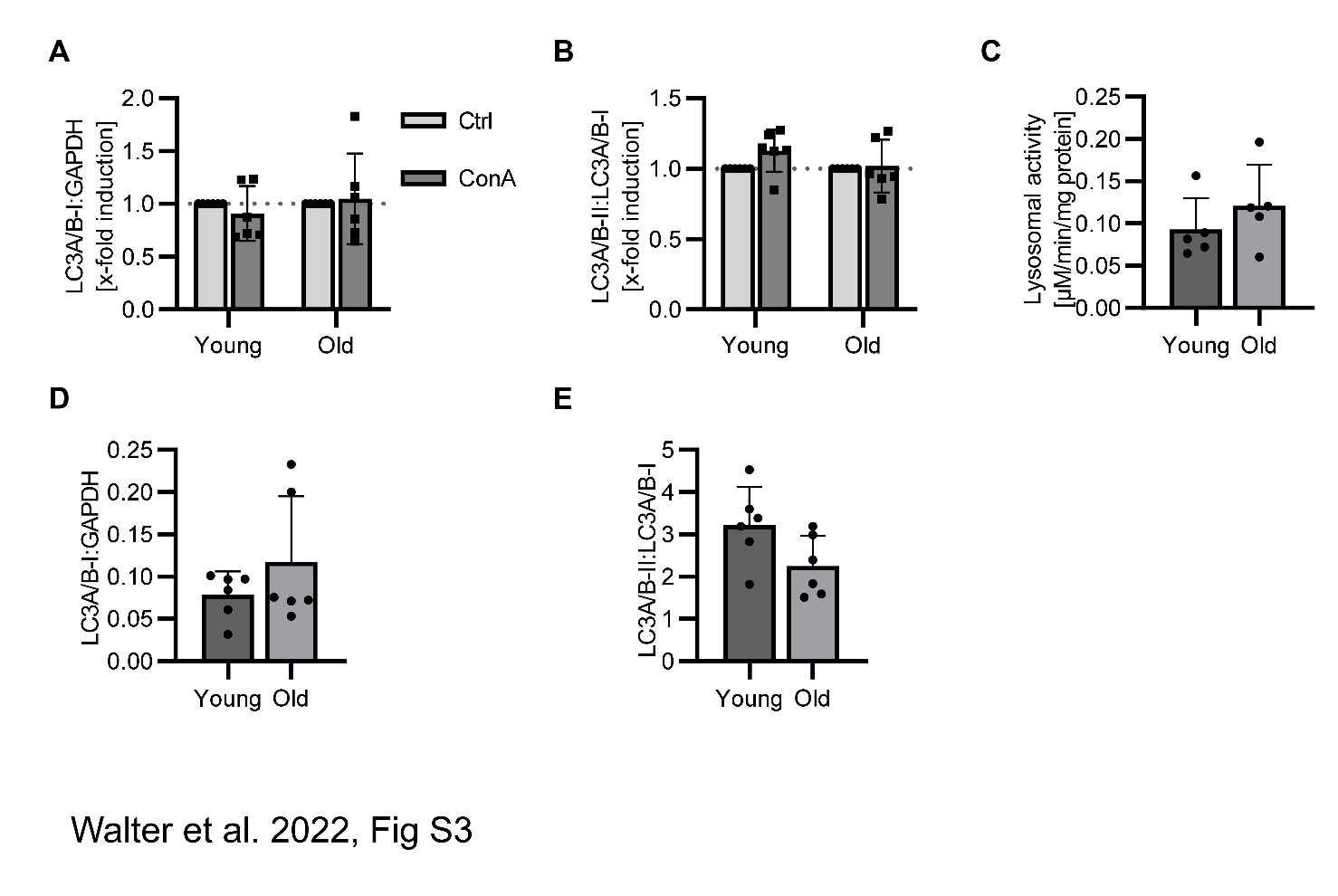


**Supplementary Figure 3:** Aging is not changing lysosomal activity nor LC3A/B levels in murine PBMCs. Murine PBMCs were isolated from young (21-22 w) and old (105-106 w) mice and incubated with or without ConA for 2 h at 37°C (n=5-6). (A)-(D) LC3A/B-I and LC3A/B-II:LC3A/B ratio protein levels were determined by western blot and normalized to GAPDH. (A)-(B) LC3A/B protein levels remained unaffected by ConA incubation and (C)-(D) control protein levels were not altered by age. (E) Lysosomal cathepsin activity did not differ between young and old murine PBMCs. Data represent mean ± SD. Statistical significance was tested with students t-test.


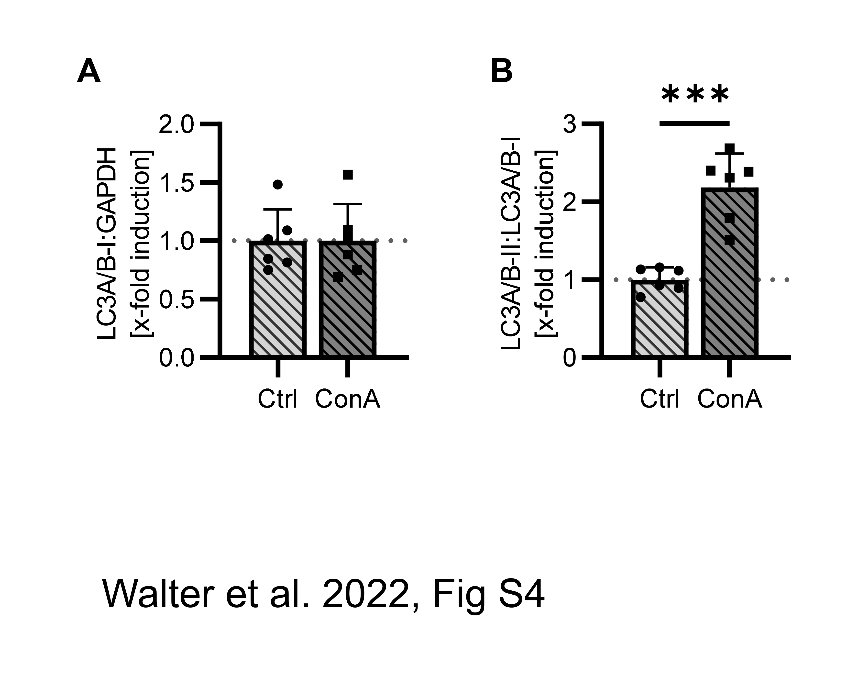


**Supplementary Figure 4:** LC3A/B ratio but not LC3A/B-I protein levels were enhanced by ConA treatment in human PBMCs. PBMCs were isolated from human blood and incubated for 2 h at 37°C with 100 nM ConA (n=5-6). LC3A/B protein levels were measured by western blot and normalized to GAPDH. (A) ConA treatment did not affect LC3A/B-I levels and (B) increased LC3A/B-II:LC3A/B-I protein levels in human PBMCs. Data represent mean ± SD. Statistical significance was tested with students t-test and given as follows: ***p≤0.001.
